# Lower Pill Burden and Once-Daily Antiretroviral Treatment Regimens for HIV Infection: A Meta-Analysis of Randomized Controlled Trials

**DOI:** 10.1093/cid/ciu046

**Published:** 2014-01-22

**Authors:** Jean B. Nachega, Jean-Jacques Parienti, Olalekan A. Uthman, Robert Gross, David W. Dowdy, Paul E. Sax, Joel E. Gallant, Michael J. Mugavero, Edward J. Mills, Thomas P. Giordano

**Affiliations:** 1 Department of Epidemiology, Pittsburgh University Graduate School of Public Health, Pennsylvania; 2 Departments of Epidemiology and International Health, Johns Hopkins Bloomberg School of Public Health, Baltimore, Maryland; 3 Department of Medicine,; 4 Centre for Infectious Diseases, Faculty of Medicine & Health Sciences, Stellenbosch University, Cape Town, South Africa; 5 Department of Biostatistics and Clinical Research, Côte de Nacre University, Côte de Nacre Teaching Hospital; 6 Faculté de Médecine, Université de Caen Basse-Normandie, EA 4655 Risque Microbien, Caen, France; 7 Division of Health Sciences, Warwick-Centre for Applied Health Research and Delivery (WCARHD), Warwick Medical School, The University of Warwick, Coventry; 8 Liverpool School of Tropical Medicine, International Health Group, United Kingdom; 9 Centre for Evidence-based Health Care, Faculty of Health Sciences, Stellenbosch University, Cape Town, South Africa; 10 Perelman School of Medicine, and Philadelphia Veterans Affairs Medical Center, University of Pennsylvania; 11 Brigham and Women's Hospital, Harvard Medical School, Boston, Massachusetts; 12 Southwest CARE Center, Santa Fe, New Mexico; 13 University of Alabama at Birmingham; 14 Faculty of Health Sciences, University of Ottawa, Ontario, Canada; 15 Department of Medicine, Baylor College of Medicine, and The Center for Innovations in Quality, Effectiveness and Safety, Michael E. DeBakey VA Medical Center, Houston, Texas

**Keywords:** randomized controlled trials, ART, fixed-dose combination, once-daily, twice-daily

## Abstract

***Background.*** Contemporary antiretroviral treatment regimens are simpler than in the past, with lower pill burden and once-daily dosing frequency common. We performed a meta-analysis of randomized controlled trials (RCTs) to investigate the impact of pill burden and once-daily vs twice-daily dosing on ART adherence and virological outcomes.

***Methods.*** A literature search of 4 electronic databases through 31 March 2013 was used. RCTs comparing once-daily vs twice-daily ART regimens that also reported on adherence and virological suppression were included. Study design, study population characteristics, intervention, outcome measures, and study quality were extracted. Study quality was rated using the Cochrane risk-of-bias tool.

***Results.*** Nineteen studies met our inclusion criteria (N = 6312 adult patients). Higher pill burden was associated with both lower adherence rates (*P* = .004) and worse virological suppression (*P* < .0001) in both once-daily and twice-daily subgroups, although the association with adherence in the once-daily subgroup was not statistically significant. The average adherence was modestly higher in once-daily regimens than twice-daily regimens (weighted mean difference = 2.55%; 95% confidence interval [CI], 1.23 to 3.87; *P* = .0002). Patients on once-daily regimens did not achieve virological suppression more frequently than patients on twice-daily regimens (relative risk [RR] = 1.01; 95% CI, 0.99 to 1.03; *P* = .50). Both adherence and viral load suppression decreased over time, but adherence decreased less with once-daily dosing than with twice-daily dosing.

***Conclusions.*** Lower pill burden was associated with both better adherence and virological suppression. Adherence, but not virological suppression, was slightly better with once- vs twice-daily regimens.

## Abstract (precis)

Once-daily compared with twice-daily antiretroviral therapy regimens increased adherence; however, the difference was modest and not associated with a difference in virological suppression. In addition, higher pill burden was associated with lower rates of virological suppression, whether once- or twice-daily regimens.

Among human immunodeficiency virus (HIV)–infected patients, adherence to antiretroviral therapy (ART) is a primary determinant of virological suppression, disease progression, and death [1–3]. ART regimens are now simpler than they were in the past, with lower pill burden and dosing frequency; they have also become less toxic and better tolerated [4]. In 2006, tenofovir–emtricitabine–efavirenz became the first approved branded, fixed-dose, single-tablet regimen (STR) [5, 6]. Two other STRs were subsequently approved by the US Food and Drug Administration: tenofovir–emtricitabine–rilpivirine and tenofovir–emtricitabine–elvitegravir–cobicistat [7, 8], both of which are currently recommended by the US Department of Health and Human Services [9].

Little is known about the impact of once- vs twice-daily ART and pill burden on adherence and virological outcomes. Indeed, in some patients with suboptimal adherence and/or virological failure, reducing the pill burden may be more important than switching from a twice-daily regimen to a once-daily regimen. Furthermore, governments, third-party payers, and HIV programs may prefer the use of non-coformulated ART generics because they are less expensive than brand name STRs. Therefore, as more generics become available, there is the potential for a paradoxical “desimplification,” with movement away from STR regimens [10, 11].

A 2009 meta-analysis by Parienti and colleagues of 11 randomized trials reported that ART adherence rates were significantly better with once-daily than with twice-daily regimens [12], with a modest effect that was more pronounced at the time of treatment initiation and was not observed in ART-experienced patients. However, that study did not find a significant effect of once-daily vs twice-daily regimens on virological outcome, possibly because of insufficient statistical power [13]. Since 2009, more randomized clinical trials comparing once- vs twice-daily regimens have been published, allowing a pooled meta-analysis with greater power to reinvestigate this question as well as the impact of pill burden [14–26]. Also, these more recent trials investigated better-tolerated, more contemporary regimens that are currently in wide clinical use.

Thus, we conducted an updated meta-analysis to evaluate the impact of pill burden and once- vs twice-daily ART on adherence as well as virological outcomes in both ART-naive and -experienced HIV-infected adults.

## METHODS

### Protocol and Registration

The study background, rationale, and methods were specified in advance and documented in a protocol that was published in the PROSPERO register (CRD42012002515).

### Inclusion Criteria

We included only randomized controlled trials (RCTs) that compared once-daily vs twice-daily regimens in either ART-naive or -experienced patients with objective measures of adherence and measures of virological outcomes.

### Search Strategy

We systematically searched the following databases from their inception until 31 March 2013 (including those years searched by the Parienti meta-analysis): Cochrane CENTRAL, PubMed, Google scholar, and Web of Science. Our search terms included the following: “HIV,” “treatment simplification,” “co-formulation,” “fixed-dose combination,” “QD,” “twice-daily,” “once-daily,” “adherence,” “HAART,” “ART,” “cART,” and “patient preference.” We also searched abstracts from major HIV/AIDS and infectious diseases conferences (from 2008 onward) including Conference on Retrovirus and Opportunistic Infections, International AIDS Conference, International AIDS Society Conference on HIV Treatment, Pathogenesis and Prevention, International Conference on Antimicrobial Agents and Chemotherapy, and Infectious Diseases Society of America Conference. In addition, the bibliographies of relevant review articles, metaanalyses, and selected articles were examined for pertinent studies.

### Study Selection

We evaluated each identified study using the following predetermined selection criteria: open-label RCTs of HIV-infected subjects either ART naive or ART experienced that compared once-daily ART regimens with any twice-daily antiretroviral regimens and assessed both adherence (using objective measures, such as pill count or medication event monitoring system [MEMS]) and viral suppression (percentage of subjects with HIV-1 RNA levels < 50 copies/mL or < 200 copies/mL in the intent-to-treat, missing-equals-failure analysis). Placebo-controlled, blinded trials were excluded because the regimen frequency was identical for the comparator arms (to maintain blinding) and, therefore, the impact of the placebo on adherence could not be measured. We chose to exclude trials that used self-reported adherence as the patients are more likely to overestimate adherence due to social desirability and typically these trials do not reflect true variability in adherence due to a ceiling effect [27–30].

### Validity Assessment

We used the Cochrane Collaboration's tool for assessing the risk of bias for quality assessment of the included studies [31]. The studies were graded based on the following: sequence generation, blinding of outcome assessor, incomplete outcome data, selective outcome reporting, and other sources of bias. The other sources of bias considered whether the analysis was intention-to-treat. We summarized the global assessment for each trial as low risk, unclear, or high risk of bias.

### Data Extraction

Three reviewers (O. A. U., J. J. P., and J. B. N.) independently evaluated the eligibility and methodological quality of studies obtained from the literature search. These same reviewers also independently extracted and compared the data. For each identified study that met the selection criteria, details on study design, study population characteristics, intervention, outcome measures, and study quality were extracted. Discrepancies were resolved by consensus through discussion.

### Summary Measures

The primary measures of treatment effects were weighted mean difference (WMD) with 95% confidence interval (CI) for adherence to treatment and relative risk (RR) with 95% CI for virological suppression. We used the following methods to compute effect sizes, when incompletely reported: contact with the corresponding author; estimation of the standard deviation (SD) on the basis of the sample size, median, and range as suggested by Hozo and colleagues [32] or on the basis of the sample size and *P* value; and imputation of the SD reported in similar studies.

### Statistical Analysis

The Spearman rank correlation coefficient (rho) was used to examine the associations between regimen pill burden (daily number of tablets), length of follow-up period, adherence rates, and virological response.

We used DerSimonian and Laird [33] random effect models to synthesize results across studies due to anticipated heterogeneity resulting from the differences in methodology, population, and ART regimen. Between-study heterogeneity was assessed using the *I^2^* statistic, which reports the percentage of total variation across studies due to heterogeneity rather than chance [34, 35]. Based on a significant interaction previously found in the meta-analysis by Parienti et al [12], subgroup analyses were prespecified to explore the reasons for heterogeneity. These were based on patient characteristics at baseline, including the following: treatment-naive individuals initiating their first regimens of ART, treatment-experienced individuals with virological suppression, and treatment-experienced individuals with treatment failure (ie, lack of virological suppression).

We examined the reliability and conclusiveness of the available evidence using a trial sequential analysis (TSA) [36–39] and the sample size required for a reliable and conclusive meta-analysis. Therefore, we calculated the sample size (ie, the heterogeneity-corrected optimal information size [HOIS]) required to detect or reject a once-daily regimen intervention effect of minimal relevant difference of 2 percentage points in mean adherence and a 10% RR difference in viral suppression. We then used the HOIS to construct Lan-DeMets sequential monitoring boundaries for our cumulative metaanalyses analogous to interim monitoring in an RCT [36–39]. We conducted the TSA with the intention of maintaining an overall 5% risk of a type I error and 20% risk of a type II error.

This review was performed according to the PRISMA recommendations for meta-analyses of RCTs [40]. Stata 12 (Stata Corporation, College Station, TX) and Review Manager 5.2 software (http://ims.cochrane.org/revman) were used for meta-analysis; Trial Sequential Analysis Software, version 0.9 beta (www.ctu.dk/tsa), was used for the trial sequential analyses.

## RESULTS

### Study Selection and Characteristics

**Figure 1.**
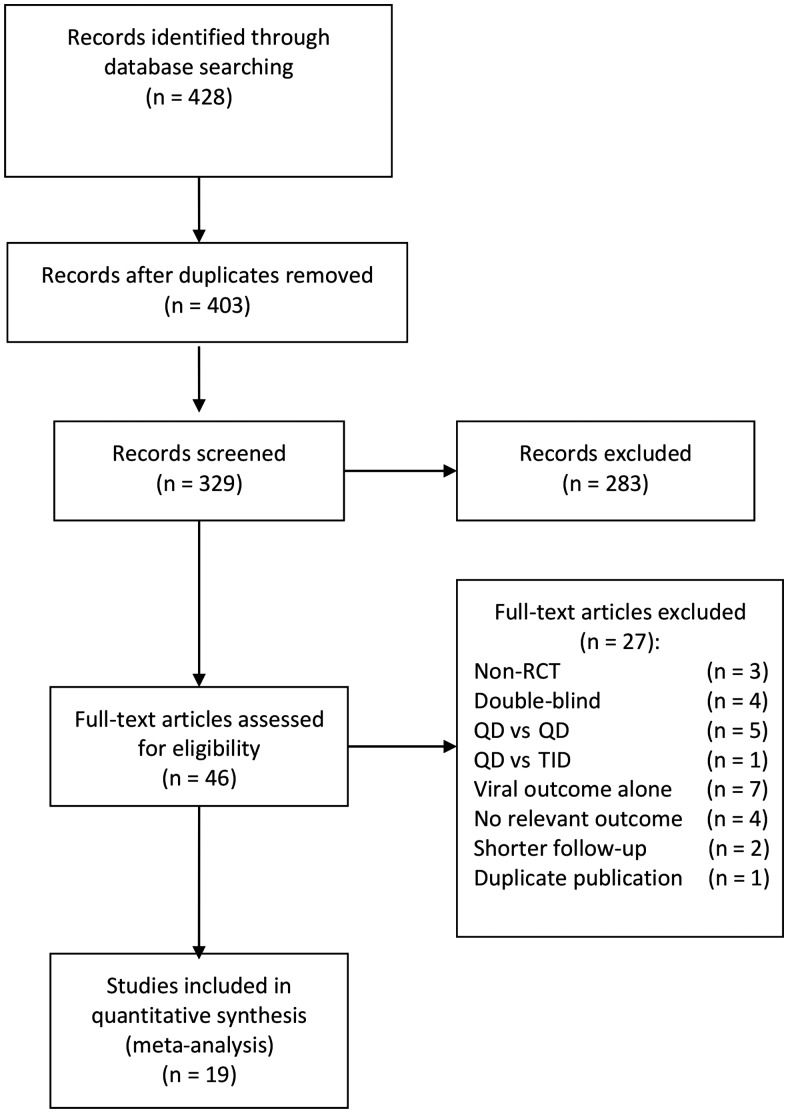
Study selection flow diagram. Abbreviations: QD, once daily; RCT, randomized controlled trial; TID, three times a day.

The literature search yielded 428 articles (Figure 1). After review, 46 articles were selected for critical reading. Of the 46 articles, 27 did not meet the inclusion criteria and were excluded. Nineteen studies [5, 17–19, 21, 22, 24, 41–49, 51–53 ] with useable outcome data involving 6312 individuals met the inclusion criteria and were included. Table 1 shows the characteristics of the included studies. The studies were published between 2004 and 2011; 11 studies with 3029 patients were included in the earlier meta-analysis [12] and 8 additional studies with 3283 patients were identified. Most studies (18/19; 95%) were published in peer-reviewed journals. Seven studies (37%) included treatment-naive patients, 9 (47%) evaluated treatment-experienced patients with suppressed viral loads, and 3 (16%) evaluated treatment-experienced patients with unsuppressed viral loads. The median duration of follow-up was 48 weeks (range, 4–96 weeks). Most studies (N = 17; 89%) reported both adherence and virological suppression. Eleven studies (58%) used MEMS to measure adherence, and 8 studies used pill count ratio. Supplementary Table 1 shows the characteristics of studies that were excluded from the meta-analysis, and Supplementary Table 2 shows the assessment of bias risk among the included studies.

**Table 1.** Characteristics of Studies Included in a Meta-Analysis of Once-Daily vs Twice-Daily Antiretroviral Therapy Regimens

Study	Year	Once-Daily Regimen	Twice-Daily Regimen	Population	Follow-up, weeks	Means of Assessing Adherence	Outcomes Reported	Risk of Bias
Benson [41]	2004	FTC, D4T or AZT, and an NNRTI or a PI	3TC, D4T or AZT, and an NNRTI or a PI	Experienced-controlled	48	Pill count	Both	Low
Eron [43]	2004	LPV/r and NRTIs	LPV/r and NRTIs	Treatment-naive	48	MEMS	Both	Low
Sosa [53]	2005	ABC, 3TC, and a PI or NNRTI	ABC, 3TC, and a PI or NNRTI	Experienced-controlled	48	Pill count	Both	Low
Gallant [5]	2006	TDF, FTC, and EFV	AZT, 3TC, and EFV	Treatment-naive	48	Pill count	Both	Low
Kubota [44]	2006	ABC, 3TC, and a third agent	ABC, 3TC, and a third agent	Treatment-naive	12	MEMS	Adherence	Low
LaMarca [45]	2006	ABC/3TC (FDC) + TDF + New NNRTI or PI	ABC + 3TC + TDF + new NNRTI or PI	Experienced-failing	48	Pill count	Both	Low
Portsmouth [51]	2006	D4T XR, 3TC, and EFV	D4T or AZT, 3TC, and EFV	Experienced-controlled	24	MEMS	Both	Low
Ruane [52]	2006	AZT, 3TC, ABC and EFV	AZT, 3TC, ABC and EFV	Experienced-controlled	24	MEMS	Both	Low
Molina [48]	2007	LPV/r, TDF and FTC	LPV/r, TDF and FTC	Treatment-naïve	96	MEMS	Both	Low
Parienti [49]	2007	NVP and NRTIs	NVP and NRTIs	Experienced-controlled	16	MEMS	Both	Low
Boyle [42]	2008	D4T XR, 3TC, and EFV	NRTIs and PI or NNRTI	Experienced-controlled	48	MEMS	Both	Low
Maitland [46]	2008	ABC and 3TC	ABC and 3TC	Experienced-controlled	4	MEMS	Both	Low
Molina [47]	2008	ATV/r plus TDF-FTC	LPV/r plus TDF-FTC	Treatment-naïve	48	Pill count	Both	High
Campo [24]	2010	EFV plus NRTIs	EFV plus NRTIs	Experienced-controlled	48	Pill count	Both	Low
Flexner [22]	2010	LPV/r and NRTIs	LPV/r and NRTIs	Treatment-naïve	48	MEMS	Both	Low
Gonzalez-Garcia [21]	2010	LPV/r, FTC, and TDF	LPV/r, FTC, and TDF	Treatment-naïve	96	MEMS	Both	Low
Zajdenverg [19]	2010	LPV/r and NRTIs	LPV/r and NRTIs	Experienced-failing	48	MEMS	Both	Low
Arasteh [18]	2011	NPV XR plus NRTIs	NPV IR plus NRTIs	Experienced-controlled	24	Pill count	Both	Low
Cahn [17]	2011	DRV/r and NRTIs	DRV/r and NRTIs	Experienced-failing	48	Pill count	Both	Low

The generation of the allocation sequence was adequately reported in 8 studies (42%) and inadequately reported in 11 studies (58%). Potential risk of bias likely to be introduced by incomplete data was low in 16 studies (84%), unclear in 2 studies (11%), and high in 1 study [47] (imbalanced loss to follow-up). There was evidence of selective reporting in 3 studies (16%) that reported adherence alone. Most studies used intention to treat analysis (n = 18, 95%).

Abbreviations: 3TC, lamivudine; ABC, abacavir; ATV/r, atazanavir/ritonavir; AZT, zidovudine; d4T, stavudine; DRV/r, darunavir/ritonavir; EFV, efavirenz; FDC, fixed-dose combination; FTC, emtricitabine; LPV/r, lopinavir/ritonavir; MEMS, Medication Event Monitoring System; NA, not applicable; NNRTI, non-nucleoside reverse-transcriptase inhibitor; NRTIs, nucleoside reverse-transcriptase inhibitor; NVP, nevirapine; PI, protease inhibitor; TDF, tenofovir; XR, extended release.

### Pill Burden

**Figure 2.**
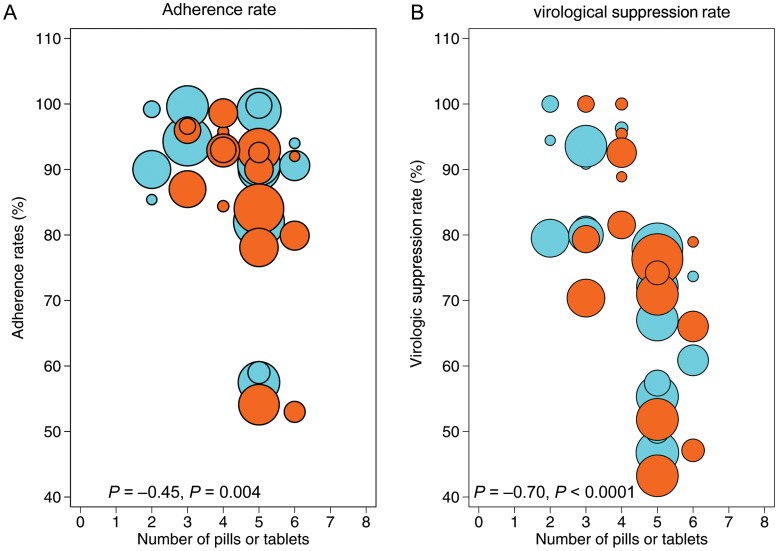
Antiretroviral therapy adherence rate, virological response, and pill burden. Area of circle is proportional to the sample size. Blue, once-daily regimens; orange, twice-daily regimens.

There was a negative and statistically significant association (Figure 2*A*) between adherence and pill burden (Spearman correlation = −0.45; 95% CI, −.67 to −.16; *P* = .004) for both once-daily and twice-daily regimens. However, when the analysis was stratified by the regimens, the association between adherence and pill burden was significant in the twice-daily regimens (Spearman correlation = −0.67; 95% CI, −.86 to −.37; *P* = .001) but not in the once-daily regimens (Spearman correlation = −0.22; 95% CI, −.60 to .25; *P* = .35). There was also a statistically significant negative association (Figure 2*B*) between pill burden and virological suppression (Spearman correlation = −0.70; 95% CI, −.84 to −.49; *P* < .0001), which was significant in both the once-daily (Spearman correlation = −0.63; 95% CI, −.85 to −.23; *P* = .005) and twice-daily subgroups (Spearman correlation = −0.75; 95% CI, −.90 to −.44; *P* = .0003).

### Once-Daily Dosing

**Figure 3.**
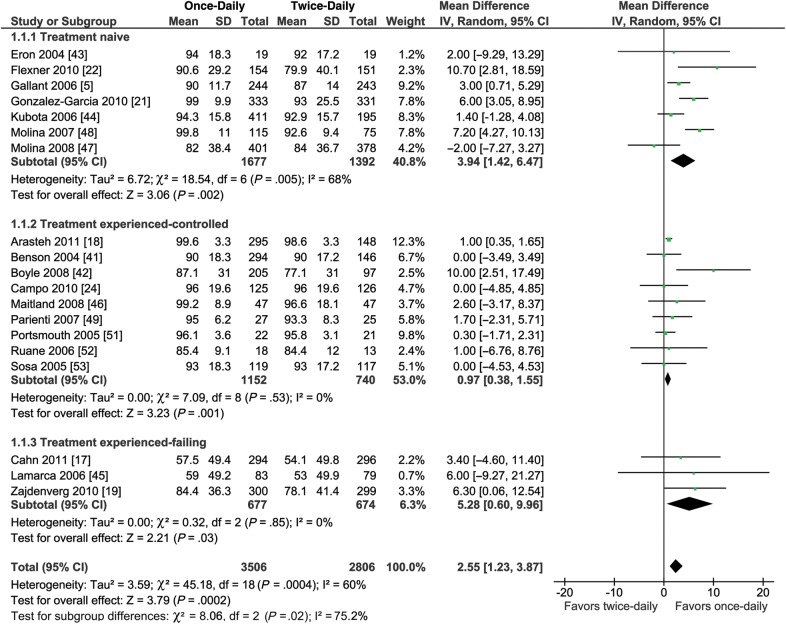
Forest plot of the effect of once-daily vs twice-daily antiretroviral regimens on the rate of adherence. Abbreviations: CI, confidence interval; IV, inverse variance; SD, standard deviation.

**Figure 4.**
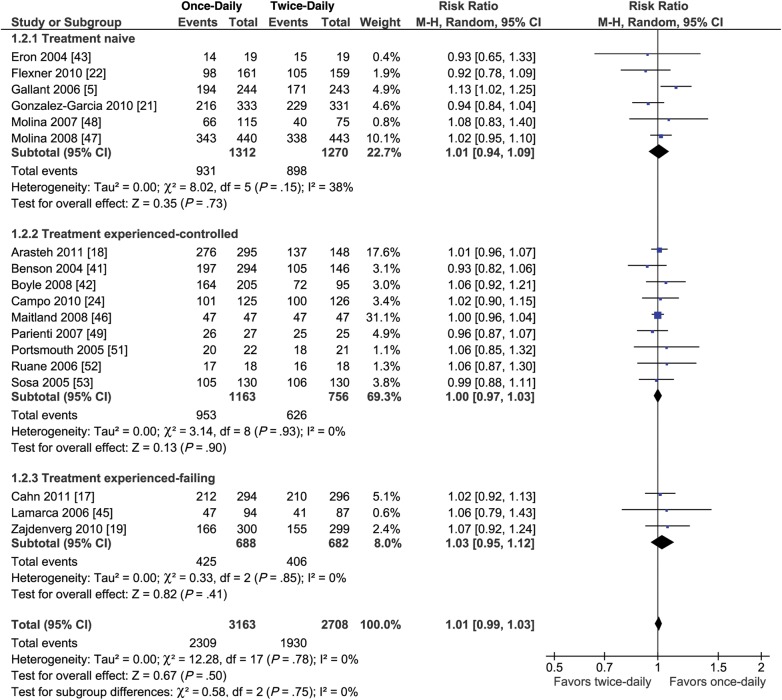
Forest plot of the effect of once-daily vs twice-daily antiretroviral regimens on virologic suppression (plasma RNA HIV level <50 or <200 copies/mL). Abbreviations: CI, confidence interval; HIV, human immunodeficiency virus; M-H, Mantel-Haenszel.

When all populations were combined, mean adherence was slightly higher among participants following once-daily regimens than those; following twice-daily regimens (WMD = 2.55%; 95% CI, 1.23–3.87; *P* = .0002; Figure 3). The trial sequential analysis demonstrated that for the regimens evaluated, the meta-analysis was conclusive (Supplementary Figure 1). In prespecified subgroup analyses, the greater average adherence with once-daily vs twice-daily dosing was more pronounced in treatment-naive patients (WMD = 3.94%; 95% CI, 1.42–6.47; *P* = .002; Figure 3) and treatment-experienced patients with virological failure switching to once-daily dosing (WMD = 5.28%; 95% CI, .60–9.96; *P* = 0.03; Figure 3) than in treatment-experienced patients who switched (for simplification/convenience) when their viral load was suppressed (WMD = 0.97%; 95% CI, .38–1.55; *P* = 0.53, Figure 3). These differences between subgroups were statistically significant (*P* = .02 for interaction). There was no significant difference in virological suppression among patients following once-daily vs twice-daily regimens (RR = 1.01; 95% CI, .98–1.03; *P* = .57; *I*^2^ = 0%, Figure 4). Trial sequential analysis suggested that as of 2007 (after the ninth trial), sufficient evidence had accrued to demonstrate that the likelihood of finding a treatment effect was too low to justify further data collection. We therefore conclude that any possible intervention effect of once-daily regimens vs twice-daily regimens is lower than a 10% RR reduction in virological suppression (the prespecified threshold; Supplementary Figure 2). Furthermore, there was no significant difference between once- and twice-daily regimens in virological suppression in the treatment-naive or -experienced subgroups (Figure 4).

### Duration of Follow-up and Treatment Effects

Adherence declined significantly over time (Spearman correlation = −0.41; 95% CI, −.64 to −.11; *P* = .009; Supplementary Figure 3*A*). When the analysis was stratified by dosing regimens, twice-daily remained statistically significant (Spearman correlation = −0.50; 95% CI, −.80 to −.03; *P* = .04), whereas the once-daily was not (Spearman correlation = −0.368; 95% CI, .697 to .088; *P* = .110). Similarly, there was a significant negative association (Supplementary Figure 3*B*) between virological suppression and duration of follow-up (Spearman correlation = −0.700; 95% CI, −.836 to −.482; *P*< .0001), such that virological suppression declined with longer follow-up. The associations were similar to the overall for both twice-daily (Spearman correlation = −0.692; 95% CI, −.876 to −.333; *P* = .002) and once-daily (Spearman correlation = −0.709; 95% CI, −.833 to −.362; *P* = .001) regimens.

Of note, in a post hoc sensitivity analysis, inclusion of studies with self-reported adherence or virological outcomes only did not materially change our results (data not shown).

## DISCUSSION

This meta-analysis of 19 RCTs which included 6312 patients found that higher pill burden was associated with both lower adherence and worse virological suppression in both twice-daily and once-daily subgroups. In addition, adherence was higher with once-daily ART regimens than with twice-daily regimens when adherence was measured objectively using pill counts and/or MEMS caps. However, this difference was minimal and did not translate into better treatment outcomes. Furthermore, the greater adherence with once-daily dosing was only statistically significant in treatment-naive individuals and in those who switched from twice- to once-daily dosing with virological failure. Adherence did not increase among treatment-experienced patients who switched from twice- to once-daily dosing while virologically suppressed; adherence was likely high in these patients prior to the switch. Both adherence and virological suppression decreased with longer follow-up, but the adherence decrease was less pronounced with once-daily dosing than with twice-daily dosing.

Interestingly, none of the included randomized trials directly evaluated the effect of an STR, which we consider an unanswered question for further research. However, in our study, there was a significant negative association between pill burden and virological suppression, suggesting that regimen simplification with STRs may be helpful in select situations. One small observational study conducted among marginally housed individuals and 2 large observational studies conducted found better adherence with STRs (compared with all other regimens, whether once daily or twice daily) [55, 56, 57], while 2 other observational studies found no difference between STRs and other once-daily regimens among patients starting ART [58] or among those who were switched from STR to multitablet regimens for reasons of cost [59].

There are several possible explanations for the apparent lack of impact of once- vs twice-daily dosing on virological outcomes. First, the impact of once-daily dosing on adherence was relatively small (2.5% absolute increase in adherence); this was possibly too small to result in a clinically meaningful difference in virological suppression. Second, a substantial number of the trials included in this meta-analysis were of relatively short duration. Moreover, volunteers for clinical trials are likely to be more adherent than their counterparts managed in routine clinical practice, and there may be more resources available to support adherence in clinical trial settings [60]. For these reasons, the difference in virological suppression that we found between once- and twice-daily ART regimens may be underestimated.

These results have several important practical implications. Currently, as all recommended regimens are highly potent, ART combinations should be selected based on factors such as tolerability, potential drug interactions, patient preference for dosing frequency, and pill burden, as well as structural factors (eg, cost, drug availability, access to care, insurance coverage) [61]. Efforts to improve and sustain adherence should not be limited to regimen simplification, but consideration should be given to proven evidence-based interventions to improve adherence such as social support [62], adherence support toolkits (eg, pillbox organizers) [63], use of cell phone and/or text messages, treatment supporters, and other targeted interventions when necessary [64–68].

In a mathematical simulation, Walensky and colleagues showed that the future use of a once-daily regimen that includes generic efavirenz plus generic lamivudine plus branded tenofovir in the United States could yield savings of almost $1 billion per year to HIV programs [69]. Our results suggest that these savings may be counterbalanced, in part, by worse virological outcomes if an increase in pill burden is required. However, no study, including ours, was specifically designed to directly investigate the impact of desimplification involving switching patients from once-daily STR to once-daily ART regimens containing multiple tablets. Further research is urgently needed to address this question.

Our study has several strengths. We performed a comprehensive search of several databases and sources to identify eligible RCTs that provide the highest quality of evidence. Three authors independently evaluated each study for inclusion and data extraction. Furthermore, we performed a trial sequential analysis; this is an efficient decision-making tool that is used to establish whether firm evidence of effect has been obtained [70]. Regarding limitations, most studies were of good quality with a low risk of bias. However, to the extent that their evidence was potentially biased, those biases are mirrored in our analyses. Notably, the likelihood of attrition bias, with a systematic difference between the 2 regimens in withdrawal rates, was very high in 1 study. While there was no evidence of heterogeneity in assessing virological suppression, the level of heterogeneity between studies in assessing adherence rates was high (I^2^ > 50%). Also, by focusing on once-daily vs twice-daily dosing, our analysis may have masked regimen-specific effects (eg, differences in toxicity) that have little to do with the frequency of dosing. Finally, the impact of regimen frequency and pill burden on adherence and virological outcomes in RCTs may not necessarily generalize to desimplification, in which patients may perceive that their regimen has been reduced in quality. Such a change could adversely affect adherence and/or treatment outcome, and, as noted above, specific studies to investigate this question are needed.

In this meta-analysis of 19 RCTs, we confirmed that once-daily ART regimens increased adherence when compared with twice-daily regimens, but the difference was modest and not associated with a difference in virological suppression. Importantly, we found that higher pill burden was associated with lower rates of virological suppression regardless of dosing frequency. The nonlinear correlation between pill burden and adherence or virological suppression suggests that, while ART desimplification from once-daily STRs to once-daily multitablet regimens may have adverse effects on virological outcomes, separating out STRs and/or fixed-dose combinations into their constituents is not likely to have a major detrimental impact on virological outcomes (provided that the overall pill burden does not increase dramatically). Nevertheless, further research is needed to directly investigate the impact of such a switch, in particular among patients who are virologically suppressed at baseline. In the meantime, our results suggest that pill burden should be a consideration in the selection of an antiretroviral regimen, independent of dosing frequency.

## Supplementary Data

Supplementary materials are available at *Clinical Infectious Diseases* online (http://cid.oxfordjournals.org). Supplementary materials consist of data provided by the author that are published to benefit the reader. The posted materials are not copyedited. The contents of all supplementary data are the sole responsibility of the authors. Questions or messages regarding errors should be addressed to the author.

Supplementary Data
