# Supplementary material for: Lower Pill Burden and Once-Daily Antiretroviral Treatment Regimens for HIV Infection: A Meta-Analysis of Randomized Controlled Trials
Source: Clin Infect Dis. 2014 Jan 22;58(9):1297–307. doi: 10.1093/cid/ciu046 (PMC3982838; doi:10.1093/cid/ciu046)
Supplement: Supplementary Data [file supp_58_9_1297__index.html]

Lower Pill Burden and Once-Daily Antiretroviral Treatment Regimens for HIV Infection: A Meta-Analysis of Randomized Controlled Trials — Lower Pill Burden and Once-Daily Antiretroviral Treatment Regimens for HIV Infection: A Meta-Analysis of Randomized Controlled Trials — Supplementary Data 

# Lower Pill Burden and Once-Daily Antiretroviral Treatment Regimens for HIV Infection: A Meta-Analysis of Randomized Controlled Trials

## Supplementary Data

Supplementary Data

**Files in this Data Supplement:**

- Supplementary Data - Pdf file
