# Supplementary material for: Lower Pill Burden and Once-Daily Antiretroviral Treatment Regimens for HIV Infection: A Meta-Analysis of Randomized Controlled Trials
Source: Clin Infect Dis. 2014 Jan 22;58(9):1297–307. doi: 10.1093/cid/ciu046 (PMC3982838; doi:10.1093/cid/ciu046)
Supplement: Supplementary Data [file supp_ciu046_ciu046supp_data.pdf]

Supplemental eTable 1 List of Excluded Studies

Study	Reason
Shaw et al. 2003[71]	No adherence measure (viral outcome alone)
DeJesus et al. 2004[72]	Double-blind
Podsadecki et al. 2008[50]	Same population than other study already included, Shorter follow-up
Wright et al. 2008[73]	No adherence measure (viral outcome alone)
Gathe et al. 2009[26]	No adherence measure (viral outcome alone)
King et al. 2009[25]	Same population than other study already included, Shorter follow-up
Martin et al. 2009[74]	daily versus daily
Martinez et al.2009[75]	daily versus daily
Podzamczer et al. 2009[76]	No relevant outcomes reported
Zajdenverg et al. 2010[77]	No adherence measure (viral outcome alone)
Airoldi et al. 2010[78]	Non randomized controlled trial
Bangsberg et al. 2010[55]	Non randomized controlled trial
Cooper D et al. 2010[23]	Double-blind
Cooper V et al. 2010[79]	No relevant outcomes reported
Musiime et al. 2010[80]	No adherence measure (viral outcome alone)
Nelson et al. 2010[81]	daily versus daily
Vispo et al. 2010[20]	No adherence measure (viral outcome alone)
Clumeck et al. 2011[82]	daily versus TID
Cohen et al. 2011 [83]	daily versus daily
Eron et al. 2011[16]	Double-blind
Honda et al. 2011[84]	daily versus daily
Maserati et al. 2011[15]	No adherence measure (viral outcome alone)
Molina et al. 2011[14]	Double-blind
Bunupuradah 2012[85]	No objective measure of adherence
Andersson 2013[86]	No objective measure of adherence
Reynes 2013[87]	No adherence measure (viral outcome alone)
Bonnet 2013[88]	No adherence measure (viral outcome alone)

Supplemental eTable 2: Risk of bias of included studies

Author	Publication year	Random sequence	Outcome assessors blinded	Incomplete outcome	Selective reporting	Intention to treat
Benson[41]	2004	?	−	?	+	+
Eron[43]	2004	?	−	+	−	+
Sosa[53]	2005	+	−	+	+	+
Gallant[5]	2006	+	−	+	+	+
Kubota[44]	2006	?	−	+	−	+
LaMarca[45]	2006	+	−	+	+	+
Portsmouth[51]	2006	?	−	?	+	+
Ruane[52]	2006	?	−	+	+	+
Molina[48]	2007	?	−	+	+	+
Parienti[49]	2007	?	−	+	+	+
Boyle[42]	2008	?	−	+	+	+
Maitland[46]	2008	+	−	+	+	+
Molina[47]	2008	+	−	−	+	+
Campo[24]	2010	+	−	+	+	+
Flexner[22]	2010	+	−	+	−	+
Gonzalez-Garcia[21]	2010	?	−	+	+	+
Zajdenverg[19]	2010	?	−	+	+	+
Arasteh[18]	2010	?	−	+	+	?
Cahn[17]	2011	+	−	+	+	+

+ Low-risk of bias ; − High risk of bias; ? Unclear risk of bias

Supplemental eFigure 1: Trial sequential analysis of the cumulative meta-analysis of the effect of once-daily versus twice-daily regimens for adherence rates

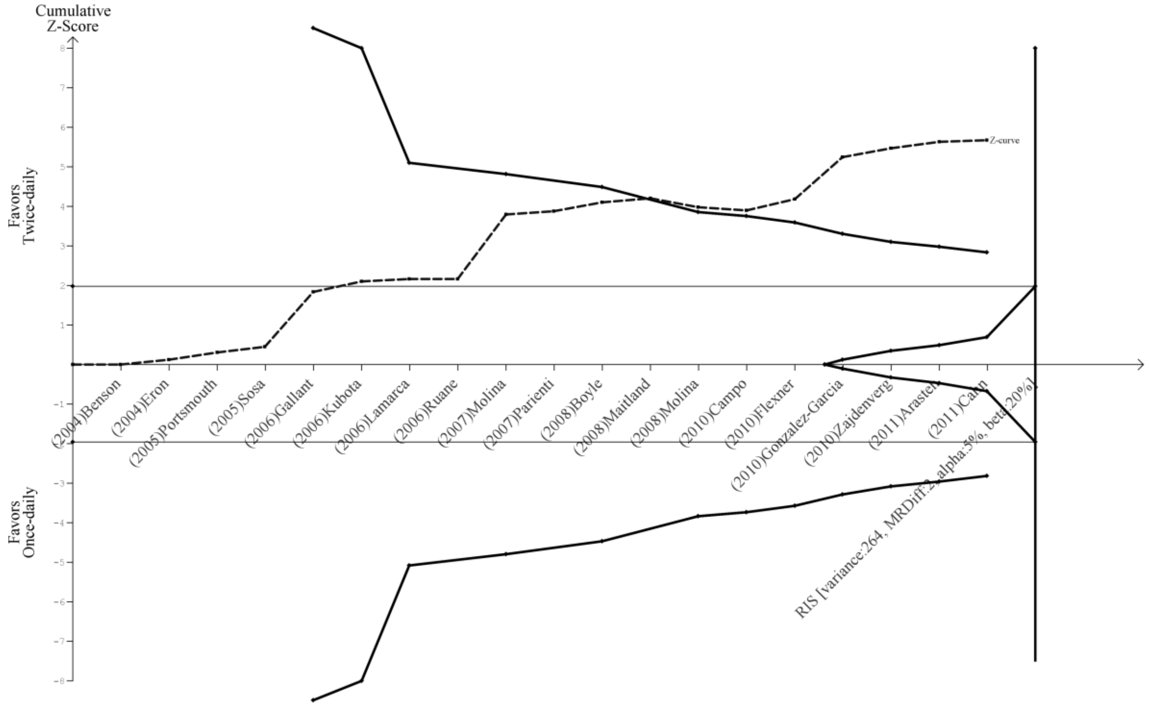

The required information size of 13,828 participants is calculated based on an intervention effect compared with no intervention of 2 points on the adherence rate, a variance of 264 on the mean difference, a risk of type I error of 5% and a power of 80%. Even with these presumptions, the cumulated Z-curve crosses the trial sequential monitoring boundaries implying that there is firm evidence for a beneficial effect of daily regimen compared with BD regimens.

Supplemental eFigure 2: Trial sequential analysis of the cumulative meta-analysis of the effect of once-daily versus twice-daily regimens for virologic suppression

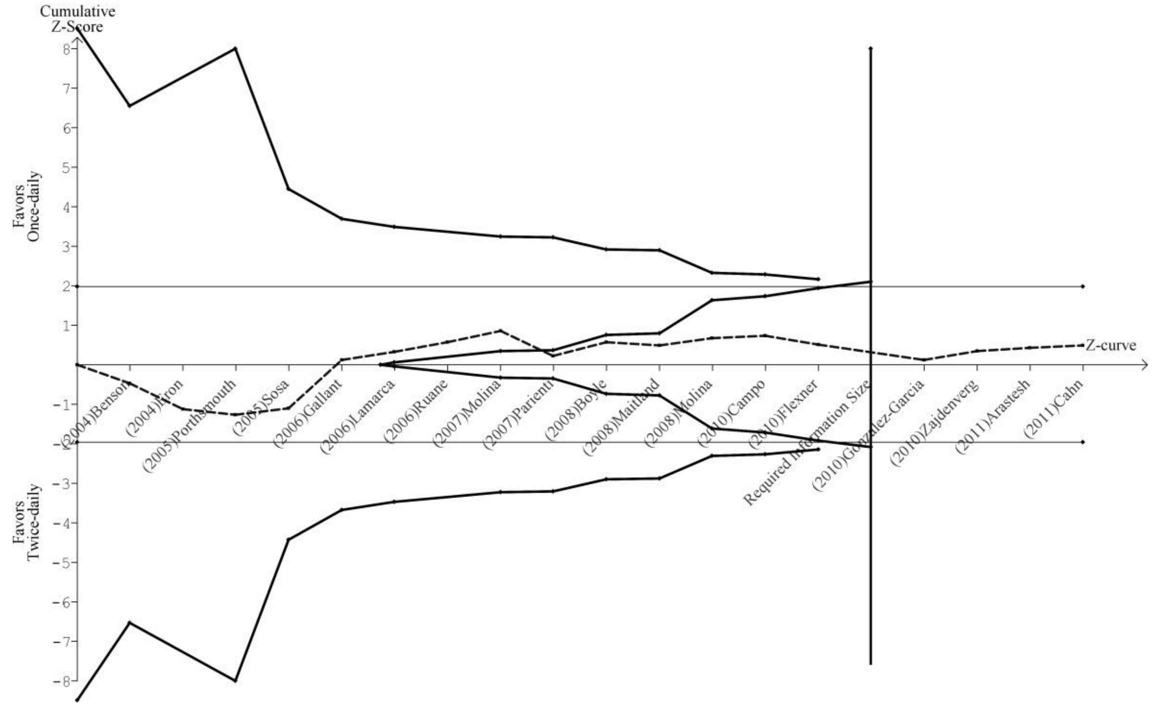

Heterogeneity adjusted required information size of 3867 participants calculated on basis of proportion of 72% with viral suppression in BD regimen, relative risk difference of 10%, a risk of type I error of 5% and a power of 80%. Cumulative Z curve does not cross solid blue trial sequential monitoring boundaries for benefit or harm, but boundaries for futility (inner wedge boundaries) are crossed. Horizontal lines illustrate traditional level of statistical significance (P=0.05)

Supplemental eFigure 3: Adherence rates, virologic response and follow-up periods

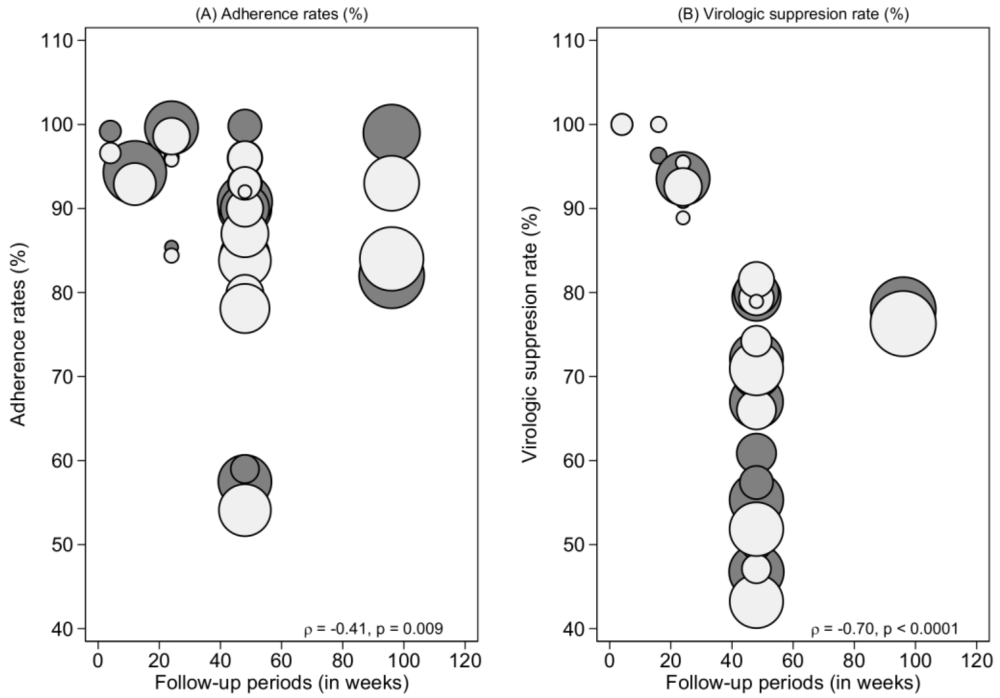

Note: Area of circle is proportional to the sample size; once-daily regimens in dark shade of grey and twice-daily regimens in light shade of grey
